# β3 adrenergic receptor as potential therapeutic target in ADPKD

**DOI:** 10.14814/phy2.15058

**Published:** 2021-10-21

**Authors:** Giorgia Schena, Monica Carmosino, Samantha Chiurlia, Laura Onuchic, Mauro Mastropasqua, Eugenio Maiorano, Francesco P. Schena, Michael J. Caplan

**Affiliations:** ^1^ Department of Cellular and Molecular Physiology Yale University School of Medicine New Haven Connecticut USA; ^2^ Department of Sciences University of Basilicata Potenza Italy; ^3^ Schena Foundation Valenzano Italy; ^4^ Department of Emergency and Organ Transplantation Section of Pathological Anatomy University of Bari Bari Italy; ^5^ Department of Emergency and Organ Transplantation Section of Nephrology University of Bari Bari Italy

**Keywords:** autosomal dominant polycystic kidney disease, G protein‐coupled receptors, SR59230A, β‐adrenergic receptors

## Abstract

Autosomal dominant polycystic kidney disease (ADPKD) disrupts renal parenchyma through progressive expansion of fluid‐filled cysts. The only approved pharmacotherapy for ADKPD involves the blockade of the vasopressin type 2 receptor (V2R). V2R is a GPCR expressed by a subset of renal tubular cells and whose activation stimulates cyclic AMP (cAMP) accumulation, which is a major driver of cyst growth. The β3‐adrenergic receptor (β3‐AR) is a GPCR expressed in most segments of the murine nephron, where it modulates cAMP production. Since sympathetic nerve activity, which leads to activation of the β3‐AR, is elevated in patients affected by ADPKD, we hypothesize that β3‐AR might constitute a novel therapeutic target. We find that administration of the selective β3‐AR antagonist SR59230A to an ADPKD mouse model (Pkd1^fl/fl^;Pax8^rtTA^;TetO‐Cre) decreases cAMP levels, producing a significant reduction in kidney/body weight ratio and a partial improvement in kidney function. Furthermore, cystic mice show significantly higher β3‐AR levels than healthy controls, suggesting a correlation between receptor expression and disease development. Finally, β3‐AR is expressed in human renal tissue and localizes to cyst‐lining epithelial cells in patients. Thus, β3‐AR is a potentially interesting target for the development of new treatments for ADPKD.

## Graphical abstract

The β3‐AR is a GPCR expressed in most segments of the murine nephron, where it modulates cAMP production. We investigate β3‐AR potential as therapeutic target in ADPKD and show that targeted β3‐AR blockade leads to a reduction in kidney/body weight through a decrease in total cAMP renal levels in a mouse model of ADPKD. Furthermore we report evidence of β3‐AR presence in biopsies from healthy and ADPKD patients.

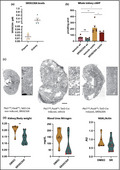

**New and Noteworthy**

We demonstrate that the β3‐AR, which like the V2R regulates cAMP signaling in the kidney, may be a promising new target for ADPKD therapy. The β3‐AR‐selective blocker SR59230A reduces cAMP levels as well as kidney size, preserves renal parenchyma integrity and produces a trend toward functional recovery in a mouse model of ADPKD. We also show that β3‐AR is expressed in human kidneys and that its expression is increased in samples from ADPKD patients

## 1 INTRODUCTION

ADPKD affects 1:400–1:1000 individuals worldwide and is the fourth leading cause of kidney failure. Its progression is characterized by formation and growth of fluid‐filled cysts arising from various nephron segments that disrupt the surrounding renal parenchyma over the course of many years, ultimately producing end stage renal disease (ESRD) in ~50% of patients (Halvorson et al., 2010). One of the pathways that has emerged as a determinant of pathogenesis involves the regulation of cellular levels of cyclic AMP (cAMP). Elevated cAMP levels in cyst‐lining cells contribute to both the cell proliferation and fluid secretion that drive cyst expansion (Belibi et al., 2004). In 2018, the FDA approved the first targeted drug for ADPKD, Tolvaptan, which acts by blocking the vasopressin type 2 receptor (V2R), a G protein‐coupled receptor (GPCR) that activates cAMP production primarily in collecting ducts (Gattone et al., 2003). There remains a pressing need for the development of additional alternative or synergistic treatments.

Kidneys are densely innervated by the sympathetic nervous system (Mompeo et al., 2016), and excessive sympathetic activity is characteristic of ADPKD (Klein et al., 2001). In fact, about 50% of ADPKD patients develop hypertension even before they experience a decline in renal function and this percentage rises to nearly 100% of the patients with ESRD. Ischemia caused by cyst compression of the renal parenchyma can also lead to sympathetic activation via increased adenosine and renin release (Klein et al., 2001). Sympathetic overactivity has been implicated in cyst progression in a rat model of ADPKD, where renal denervation partially ameliorated the phenotype (Gattone et al., 2008).

Recently, β3‐adrenergic receptor (β3‐AR) was shown to be expressed in several segments of the murine nephron, including thin and thick ascending limbs, distal convoluted tubule, and cortical collecting ducts (CCDs), whereas it was not detected in the proximal tubule and medullary collecting ducts. Selective activation of β3‐AR increases cAMP levels in isolated mouse renal tubules and activates key proteins involved in transepithelial water and solute movement (Procino et al., 2016). The physiological function and regulation of β3‐AR has been extensively studied since its discovery only three decades ago. β3‐AR is widely localized throughout the human body but is generally expressed at low levels in most tissues (Schena & Caplan, 2019). Since ligand binding by the β3‐AR is transduced at least in part through the enhanced production of cAMP (Procino et al., 2016), its activation could contribute to cAMP‐driven cystogenesis in the context of ADPKD. Based on these findings, we speculated that the β3‐AR might contribute to the effects of sympathetic over‐activity in ADPKD progression. Consistent with our hypothesis, we found that the β3‐AR is expressed at high levels in total kidney lysates from a conditional mouse model of ADPKD (Pkd1^fl/fl^; Pax8^rtTA^; TetO‐Cre). Most importantly, β3‐AR blockade via the selective antagonist SR59230A decreases cAMP levels and partially ameliorates the cystic phenotype in these mice, producing a reduction in kidney size and a trend towards preservation of renal function. Finally, we find that β3‐AR is also expressed in kidney samples from healthy and ADPKD patients, thereby demonstrating its potential relevance in the context of ADPKD therapeutic development.

## 2 METHODS

### 2.1 Mouse strain and histology

Animal protocols were approved and conducted in accordance with Yale Animal Resources Center and Institutional Animal Care and Use Committee regulations. Pdk1^fl/fl^; Pax8^rtTA^; TetO‐Cre mice and their induction regimen were previously described (Ma et al., 2013). Experiments were conducted on males. Retro‐orbital blood was collected from anesthetized mice for plasma BUN measurements (O’Brien Kidney Center at Yale). Upon sacrifice, kidney and body weights were recorded. Right kidneys were harvested and fixed in 4% PFA for histological analysis while contralateral kidneys were snap‐frozen. Images of whole‐kidney sections were obtained with a Nikon Eclipse TE2000‐U microscope under the control of MetaMorph software (Universal Imaging).

### 2.2 LC–MS/MS analysis of plasma and kidney concentrations of SR59230A

Plasma and kidney homogenates (25 µl) were processed by protein precipitation with 150 µl of acetonitrile containing 15 ng/ml of loperamide as the internal standard (IS). Calibration curve samples of SR59230A (0.001–10 µM) were prepared by spiking naïve mouse plasma and kidney homogenates with known concentrations of SR59230A and processed similarly along with study samples. Samples were centrifuged at 4800 *g* for 45 min at 4°C, and 100 µl of the supernatants were transferred to a deep well 96‐well plate containing 100 µl of LC–MS grade H_2_O. The plate was mixed by shaking for 5 min at RT, and 2 µl of the sample were injected onto the UHPLC–MS/MS system. Chromatographic separation was achieved using an Agilent Zorbax XDB‐C18 RRHD 2.1 × 50 mm column, with 0.1% acetic acid in water (mobile phase A) and 0.1% acetic acid in acetonitrile (mobile phase B) run at 0.4 ml/min as per the following gradient: 5% B for 0.3 min, 5%–90% B over 0.7 min, 95% B for 1 min, 95%–5% B over 0.1 min, and 5% B for 1.40 min. Total run time was 3.5 min. The mass spectrometer was operated in multiple reaction monitoring mode under positive ionization, with the declustering potential and collision energy settings of 131 and 35 V for SR59230A and 40 and 35 V for loperamide, respectively. The MRM transitions for SR59230A and loperamide (IS) were, respectively, m/z 325.77 → m/z 131.1 and m/z 478.14 → m/z 267.1.

### 2.3 cAMP measurement in total kidney preparations

Upon collection kidneys were immediately snap‐frozen in liquid nitrogen, ground to a fine powder in pre‐chilled ceramic mortars and then homogenized in HCl 0.1 M. Homogenates were pelleted at 600 g for 10 min at 4°C to remove debris and supernatants were processed without acetylation using an enzyme immunoassay kit according to manufacturer instructions (Enzo Life Sciences). Results are expressed in pmol/mg protein.

### 2.4 Patients and histological analysis

Normal tissue was obtained via open kidney biopsy from two cadaveric organ donors and via needle biopsy from two living kidney donors. Tissue from normal portions of nephrectomized kidneys was also obtained from three patients who underwent nephrectomy for the presence of renal cell carcinoma. Pathological renal tissue was also obtained from six ADPKD patients who underwent nephrectomy for the excision of renal cell carcinoma. Formalin‐fixed paraffin‐embedded (FFPE) renal samples were obtained from the archive of the Institute of Pathology, University of Bari, Italy. Thin sections (2 µm) of FFPE kidney specimens were deparaffinized and hydrated through xylene and graded alcohol series. After antigen retrieval in EDTA (pH 8), the endogenous peroxidase activity was quenched by incubation in a solution of 3% H_2_O_2_ for 7 min. Sections were incubated for 10′ with serum‐free protein block (Dako) and then incubated overnight at +4°C with anti‐β3‐AR (1:30, ADRB3, R&D System) followed by 1 h incubation with a biotinylated mouse secondary antibody (1:1000), which was detected by the Dako Real EnVision, Peroxidase/DAB kit (Dako). Sections were counterstained with Mayer's hematoxylin (blue). The primary antibody was omitted in negative controls. Digital images were acquired with an Olympus C‐7070 Wide Zoom camera (Olympus Imaging Corp.) and Leica/Leitz DMRB (Leica Mikroskopie & Systeme GmbH). Aperio ImageScope Software (Aperio Technologies) was used to measure the staining intensity and the percentage of positive cells using the positive pixel count v9 algorithm. All patients gave their informed consent to use remaining portions of kidney specimens for clinical studies, after diagnosis. The study was carried out according to the principles of the Declaration of Helsinki.

### 2.5 Immunofluorescence

Mouse kidney sections were co‐incubated with the appropriate primary antibodies, β3‐AR (1:100, Santa Cruz, sc‐1473), calbindin (1:50 Santa Cruz, sc‐365360), p‐NKCC2 (1:1000, R5 antibody, gift of Dr. B. Forbush, Yale University), and AQP2 (1:100, Santa Cruz, C‐17, sc‐9882), followed by 1 h RT incubation with AlexaFluor‐conjugated secondary antibodies (Life Technologies) and Hoechst nuclear staining (Invitrogen, Molecular Probes). Confocal images were obtained with a LSM780 (Carl Zeiss). Images are the product of eightfold averaging and contrast and brightness values were selected to ensure that all pixels were within the liner range.

### 2.6 Western Blot

Snap‐frozen mouse kidneys were processed and the homogenates quantified as previously reported (Ma et al., 2013). A total of 15 µg/lane of proteins in SDS‐PAGE 1× loading buffer (BioRad) with 200 mM DTT were separated on a 12% SDS–polyacrylamide gel and then electrophoretically transferred to a nitrocellulose membrane (Bio‐Rad). Membranes were incubated with primary antibody against murine β3‐AR (1:1000, Santa Cruz, sc‐50436) or NGAL (1:1000, RD Biosciences, cat#AF1857) at +4°C overnight and the appropriate infrared (IR)‐conjugated secondary, 1 h at RT (1:10000 Li‐Cor Biosciences). Membranes were visualized on an Odyssey Infrared Imager (Li‐Cor Biosciences) and quantitated using the associated software package.

### 2.7 Statistical analysis

All data are expressed as means ± SEM. Due to the relatively small sample sizes that were employed in the present preliminary assessment of the β3‐AR as a potential therapeutic target, a normal distribution could not be assumed and differences between means of the experimental groups were evaluated using the two‐tailed Mann–Whitney *U* test. We considered a *p* < 0.05 to be statistically significant. Statistical analysis was performed using GraphPad Prism 8.

## 3 RESULTS

**FIGURE 1:**
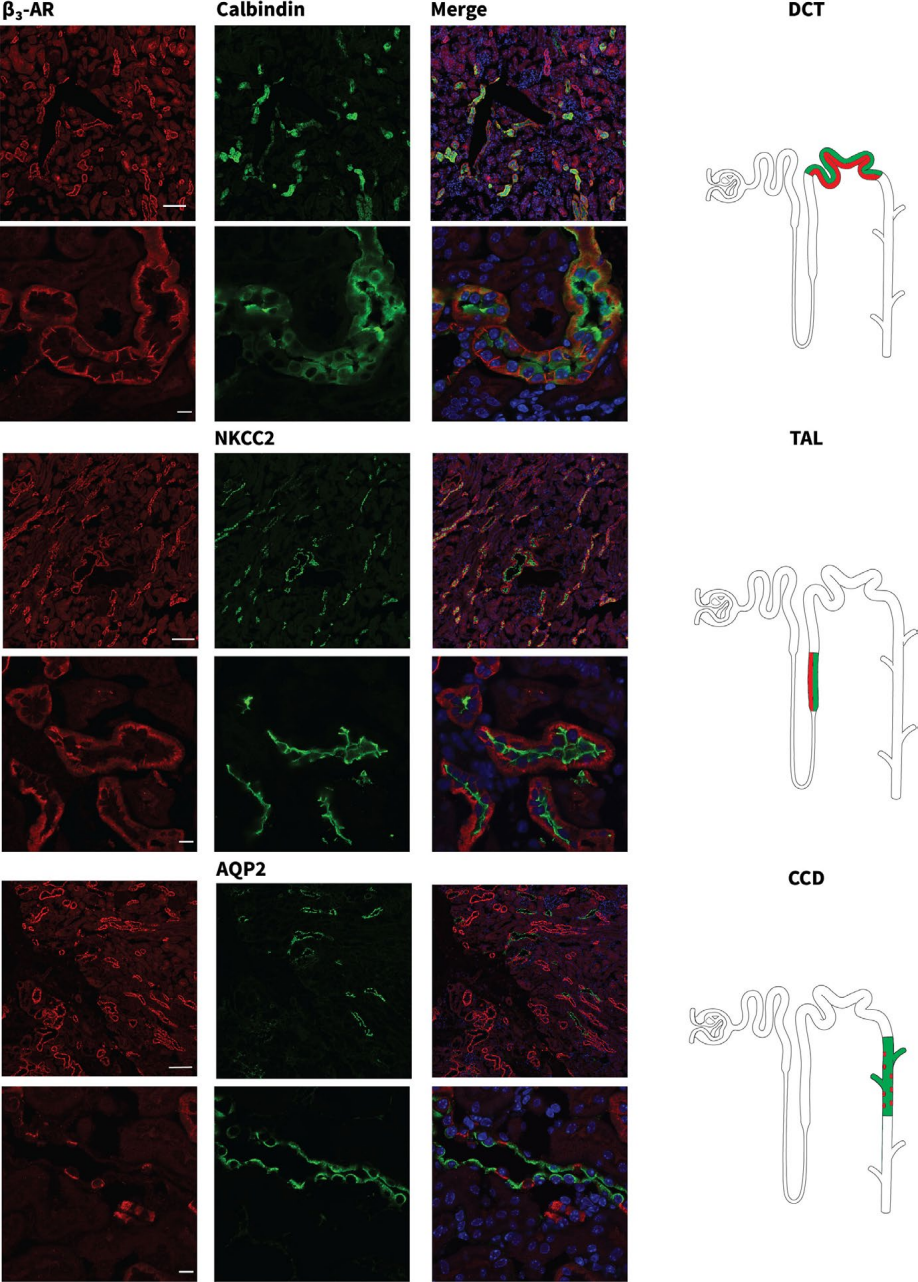
β3‐AR localizes to multiple segments of the mouse nephron. Cryosections from wild‐type (wt) mouse kidneys were stained with anti‐β3‐AR (red) and nephron segment‐specific markers (green). Calbindin for distal convoluted tubules (DCTs), Na+K+2Cl (NKCC2) co‐transporter for the thick ascending limbs (TALs) and Aquaporin 2 (AQP2) for the cortical collecting ducts (CCDs). Confocal microscopic images were acquired at two different magnifications (Upper panels, scale bar = 100 µm; Lower panels, scale bar = 10 µm). Nuclei were stained with Hoechst Nuclear Staining (blue). Positive nephron segments are depicted in the cartoons on the right and colocalization is highlighted in red and green

*β3‐AR is expressed in kidneys of a conditional mouse model of ADPKD*. We employed a previously characterized conditional knock‐out mouse model for the Pkd1 gene (Pkd1^fl/fl^; Pax8^rtTA^; TetO‐Cre) (Ma et al., 2013) which are engineered to produce doxycycline‐inducible Cre expression in renal epithelial cells. We began by assessing whether β3‐AR expression and localization in this model are consistent with data reported in the literature (Procino et al., 2016) to evaluate its potential suitability as a target in ADPKD. The distribution of β3‐AR protein was first analyzed via IF on renal sections from wild‐type (wt) mice. Using specific markers for nephron segments, including NKCC2 for thick ascending limb (TAL), Calbindin for distal convoluted tubule (DCT) and AQP2 for CCD, we confirmed that β3‐AR localizes within the basolateral plasma membrane domains of epithelial cells lining each of these renal tubule segments (Figure 1).

**FIGURE 2:**
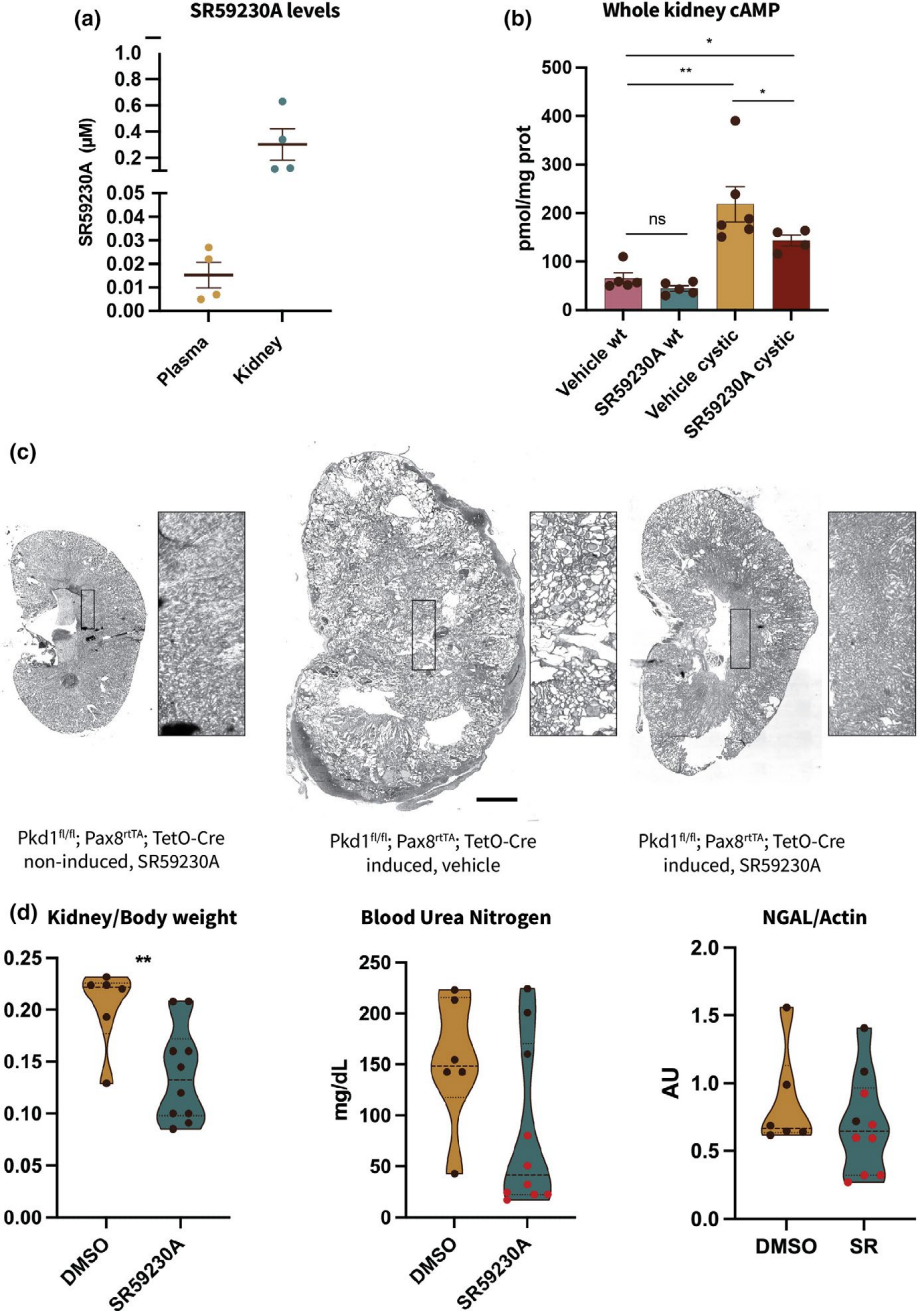
Administration of β3‐AR‐selective blocker SR59230A lowers cAMP levels and partially ameliorates the cystic phenotype in Pdk1^fl/fl^; Pax8^rtTA^; TetO‐Cre mice. (a) LC‐MS/MS analysis of SR59230A concentration in plasma and kidney tissue samples from wt male mice. (b) Measurement of whole‐kidney cAMP levels in homogenates from cystic mice shows that β3‐AR‐selective blockade lowers cAMP in SR‐treated mice as compared to vehicle‐treated littermates. **p < 0.01, *p < 0.05, ns, not significant. (c) Left to right, representative H&E images from non‐induced (left image) and induced mice (mid and right images, respectively), showing the presence of cysts in the parenchyma from cystic animals. Boxed regions are enlarged (5×) in the panel next to the kidney image. Scale bar = 1 mm. (d) Dot plots showing kidney/body weight ratio, plasma blood urea nitrogen (BUN) and NGAL protein levels normalized to actin as measured upon sacrifice at 4 weeks of treatment. The animals with the lowest levels of blood urea nitrogen are indicated with red dots in the central (blood urea nitrogen) as well as in the right‐most (NGAL/actin) panels **p < 0.01

*SR59230A administration lowers renal cAMP levels 4hrs post injection in Pdk1^fl/fl^; Pax8^rtTA^; TetO‐Cre cystic mice*. To confirm that the β3‐AR‐selective antagonist SR59230A can access renal tissue via the bloodstream, we performed mass spectrometry (MS) analysis on plasma and renal tissue samples from wt, non‐induced male mice treated with SR59230A (4 mg/kg) and euthanized after 4 h. As expected, both plasma and tissue samples contained detectable levels of SR59230A, with kidney levels (Avg 0.3 µM ± 0.2) about 20‐fold higher than those in plasma (Avg 0.015 μM ± 0.009) (Figure 2a). To test our hypothesis that selective β3‐AR blockade could potentially have an ameliorative effect on the cystic phenotype by reducing renal cAMP we measured whole‐kidney cAMP levels 4 h after SR59230A (4 mg/kg) administration in ADPKD cystic mice at 13 weeks of age that exhibited Kw/Bw ratios of 0.1 ± 0.05. It has been determined that cAMP levels increase with disease progression and renal enlargement (Ma et al., 2013; Yamaguchi et al., 1997). Thus, in order to be able to detect a change in cAMP levels we selected a time point for our analysis based on previous studies showing that an increase in cAMP levels is already evident at 13 weeks of age and 4 weeks following the activation of Cre expression (Ma et al., 2013). In addition, because cAMP levels correlate with kidney enlargement (Ma et al., 2013; Yamaguchi et al., 1997), cystic animals with comparable Kw/Bw ratios were used in the analysis. It has been determined that cAMP levels increase with disease progression and renal enlargement (Ma et al., 2013; Yamaguchi et al., 1997). Thus, in order to be able to detect a change in cAMP levels we selected a time point for our analysis based on previous studies showing that an increase in cAMP levels is already evident at 13 weeks of age and 4 weeks following the activation of Cre expression (Ma et al., 2013). In addition, because cAMP levels correlate with kidney enlargement (Ma et al., 2013; Yamaguchi et al., 1997), cystic animals with comparable Kw/Bw ratios were used in the analysis. As expected, we found that these mice have significantly higher cAMP levels than wt animals. Importantly, acute SR59230A treatment significantly lowers cAMP levels in cystic‐treated mice as compared to vehicle‐treated cystic littermates (Figure 2b).

**FIGURE 3:**
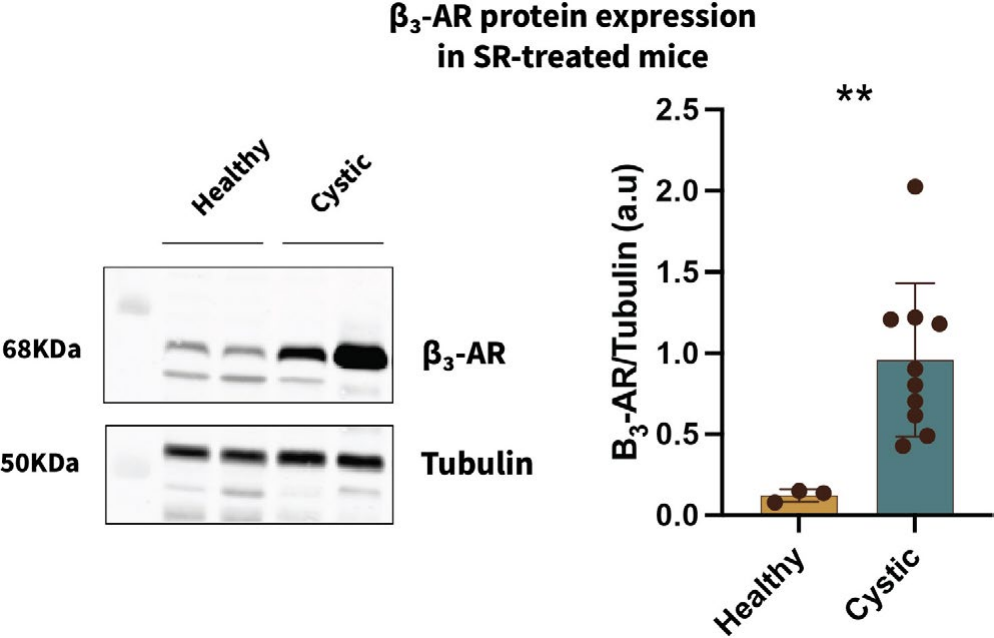
β3‐AR protein levels are significantly higher in cystic animals. β3‐AR protein levels were measured in whole‐kidney lysates from SR‐treated healthy and cystic animals and normalized to tubulin staining. β3‐AR (68 kDa) protein levels are significantly higher in cystic animals. **p < 0.01

*Chronic SR59230A treatment (4 mg/kg) ameliorates the cystic phenotype in Pdk1^fl/fl^; Pax8^rtTA^; TetO‐Cre mice*. To investigate the effect of SR59230A treatment on cyst progression we administered SR59230A for 4 weeks. Six weeks after the inactivation of Pkd1 gene, coincident with the onset of cyst development (Ma et al., 2013), male mice were randomly divided into 2 subgroups receiving SR59230A (4 mg/kg) via i.p. injection every 24 h for 4 weeks or vehicle alone (DMSO <4%). The former group also included three genetically non‐cystic healthy mice to test for untoward renal effects of the SR compound. As revealed through H&E staining, SR treatment leads to preservation of renal parenchyma (Figure 2c and insets). We observed a significant reduction in kidney/body weight ratio of SR59230A‐treated mice versus vehicle‐treated animals and a trend toward lowered BUN, indicative of improving renal function (Figure 2d). A similar trend was also observed in the levels of NGAL protein measured in kidney lysates prepared from SR‐treated animals versus DMSO‐treated controls. We found that the treated animals that manifest lower levels of BUN (red dots in Figure 2d) also showed lower levels of NGAL, thus strengthening our hypothesis that SR59230A treatment has an ameliorative effect on the cystic phenotype. Finally, we compared β3‐AR protein expression levels in total kidney lysates from treated healthy and cystic mice and found that they are significantly increased in ADPKD animals (Figure 3). This result raises the interesting possibility that ADPKD development might influence β3‐AR expression, which could in turn exacerbate cyst development by driving cAMP production.

**FIGURE 4:**
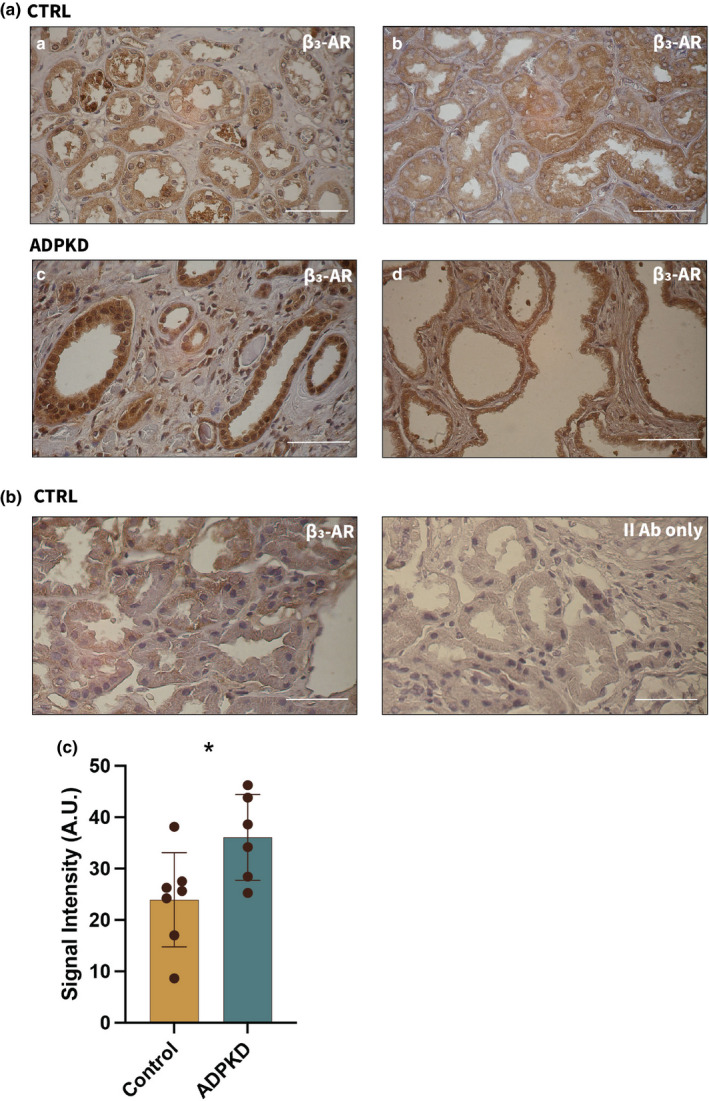
β3‐AR protein is expressed in human kidneys and it is present at higher levels in ADPKD tissue. (a) Representative images from normal (CTRL) and ADPKD kidney samples. (a) Normal living donor, (b) normal tissue surrounding renal cell carcinoma and (c, d) ADPKD patients showing β3‐AR positive staining. Scale bar = 50 µm. (b) Specificity control for anti‐β3‐AR monoclonal antibody (ADRB3, R&D System) on normal tissue (CTRL). (c) Quantification of β3‐AR in CTRL vs ADPKD tissue. *p < 0.05

*β3*‐*AR levels are elevated in kidney sections from ADPKD patients*. To determine the potential clinical relevance of our in vivo findings we quantified β3‐AR expression via immunohistochemistry (IHC) in human normal (Figure 4a, CTRL a‐b) and ADPKD (Figure 4a,c,d) kidney samples. Consistent with the mouse model, β3‐AR levels are elevated in tissue obtained from ADPKD patients as compared to healthy controls (Figure 4c). The specificity of the β3‐AR signal was confirmed by omitting the primary antibody (Figure 4b). To the best of our knowledge, this is the first report of the presence of β3‐AR in human kidney and of its elevated expression in ADPKD patients. This result strengthens the contention that the β3‐AR has the potential to be exploited as a novel and previously unexplored therapeutic target in ADPKD.

## 4 DISCUSSION

ADPKD is characterized by numerous biochemical alterations that disrupt cellular homeostasis. While this complexity provides investigators with a wealth of options to pursue in searching for potential treatments, many affected pathways are involved in processes critical for the entire organism. The approval of Tolvaptan, together with ongoing trials on the somatostatin analogue, octreotide, provide a strong rationale for pursuing additional therapies that target GPCRs and aspects of cAMP signaling (Tellman et al., 2015). Tolvaptan exerts its effect by lowering cAMP levels in renal cells that express the V2R receptor (Gattone et al., 2003). Its therapeutic utility may be limited to some extent by on‐ and off‐target side effects including polyuria, polydipsia, and liver toxicity (Chebib et al., 2018). We explored the role of the GPCR β3‐AR as a potential target for the treatment of ADPKD. While β3‐AR resembles the V2R in its ability to modulate cAMP levels in renal epithelial cells, it is worth noting that β3‐AR localizes to a broader collection of nephron segments than does the V2R (Fenton et al., 2006; Procino et al., 2016). Consistent with previous data (Procino et al., 2016), we find that β3‐AR is expressed in several nephron segments in wt mice, including TAL, DCT, and CD. Thus, a selective treatment that targets β3‐AR could potentially exert a broad effect to slow cyst development.

Hyperactivation of the sympathetic system has been reported in patients affected by ADPKD (Klein et al., 2001) and a key role for this activation is suggested by two common clinical practices: treatment of patients with angiotensin‐converting enzyme inhibitors/angiotensin II receptor blockers to control their blood pressure and renal denervation to treat kidney‐related pain (Tellman et al., 2015; Tkachenko et al., 2013).

Since sympathetic nerve activity is elevated in patients with chronic kidney disease including ADPKD (Klein et al., 2001), we hypothesize that this imbalance can lead to β3‐AR hyper‐activation and contribute to the progression of the cystic phenotype by increasing cAMP levels in those renal epithelial cells expressing the receptor. The hypothesis that β‐ARs may play a role in the pathogenesis of ADPKD has been previously proposed (Gattone et al., 2008). Interestingly, because it is not subject to rapid desensitization (Milano et al., 2018) β3‐AR may contribute to sustained elevated cAMP levels in renal cells to a greater extent than do V2R receptors.

Thus, a targeted approach that selectively inhibits β3‐AR might decrease cAMP production, thus improving the cystic phenotype. Among the few available β3‐AR‐selective antagonists we chose SR59230A, a molecule that has been previously reported to block β3‐AR in vivo (Bruno et al., 2020; Sun et al., 2020). Consistent with our hypothesis, β3‐AR‐selective blockade lowered cAMP levels in treated cystic animals as compared to vehicle‐treated cystic controls 4 h after administration. Next, to investigate SR59230A effects on the cystic phenotype, we administered SR59230A to Pkd1^fl/fl^; Pax8^rtTA^; TetO‐Cre cystic male mice daily for 4 weeks. Our results show that chronic SR59230A treatment led to a significant reduction in kidney/body weight and to a pronounced trend toward the rescue of renal function. Interestingly, β3‐AR protein quantified in total kidney lysates from healthy and cystic SR‐treated animals was markedly increased in the latter group. This result points to a possible correlation between β3‐AR expression and disease development. In fact, increased levels of receptor expression are consistent with our hypothesis that over‐stimulation of β3‐AR could contribute to disease progression in ADPKD. Moreover, elevated β3‐AR receptor levels have already been reported in the context of other pathological conditions (Calvani et al., 2015; Dincer et al., 2001).

Beta 3‐AR is widely expressed in various human tissues (Schena & Caplan, 2019) although to date its presence has not been established in the human kidney. We assessed β3‐AR expression via immunohistochemical analysis performed on sections of renal tissue obtained from healthy and ADPKD patients. Consistent with our mouse data, β3‐AR levels are higher in tissue derived from patients affected by ADPKD than in renal tissue from healthy individuals. Because β‐ARs are GPCRs whose activation can stimulate Adenylyl Cyclase to generate cAMP, if they are over‐stimulated through increased sympathetic tone they could contribute to enhanced cystogenesis in the cell types that express them. Consistent with this possibility, epinephrine has been detected in cyst fluid and elicits a significant elevation in cAMP levels in ADPKD cells from patients (Belibi et al., 2004). To date no studies have pursued ADPKD therapies directed at inhibition of adrenergic receptors, most likely due to the systemic consequences associated with blockade of the β1‐ARs and β 2‐ARs, although the presence of β1 and β2‐ARs in the kidney is limited (Boivin et al., 2001). Because β3‐AR is expressed in both the murine and human kidney, we hypothesized that receptor blockade might exert an ameliorative effect on the development of the cystic phenotype through a decrease in renal cAMP levels, which was confirmed by our in vivo experiments. Along these lines, it will be interesting to test the possibility that treatment with the SR59230A compound would synergize effectively with tolvaptan to reduce cyst growth. It is worth noting that β3‐AR blockade might be predicted to produce a modest degree of polyuria, based on the low level of polyuria described in β3‐AR KO mice (Procino et al., 2016). Because β3‐AR modulates cAMP in several nephron segments in addition to the CDs, a combination therapy employing lower doses of Tolvaptan might be more effective than Tolvaptan alone and produce fewer of the on‐target side effects that are associated with Tolvaptan monotherapy. This possibility should be tested in future studies designed to assess directly of the potential for synergistic benefits in suppressing the ADPKD phenotype that might be realized through Sr59230A/Tolvaptan combinatorial therapies. Although SR50230A administration to healthy mice did not produce short‐term renal side effects, the possible existence of off‐target side effects should be explored further. These novel discoveries must be considered in the context of the limitations of this study. While our data suggest that the observed effects result from drug‐induced changes due to exclusive engagement of the β3‐AR, it remains possible that this compound could exert influence on other β‐ARs or unrelated signaling systems. It would also be beneficial to assess the potential efficacy of SR59230A in a second ADPKD mouse model to confirm the generality of the findings that we report and the mechanism that we propose. Finally, although SR59230A shows poor selectivity for the human β3‐AR, this study constitutes the first proof of concept that β3‐AR can be successfully targeted to improve the cystic phenotype in ADPKD and supports further research into β3‐AR antagonists with a better translational profile.

## CONFLICT OF INTEREST

The authors declare no conflict of interest.

## AUTHOR CONTRIBUTIONS

G.S., M.J.C., and M.C. designed the study; G.S., S.C., and L.O. carried out experiments; G.S. analyzed the data, designed the figures, and drafted and revised the paper; M.J.C., M.C., and F.P.S. revised the paper. All authors approved the final version of the manuscript.
